# Supplementary material for: Novel Affibody Molecules Specifically Bind to SARS-CoV-2 Spike Protein and Efficiently Neutralize Delta and Omicron Variants
Source: Microbiol Spectr. 2022 Dec 13;11(1):e03562-22. doi: 10.1128/spectrum.03562-22 (PMC9927262; doi:10.1128/spectrum.03562-22)
Supplement: Supplemental file 1 — Supplemental material. Download spectrum.03562-22-s0001.pdf, PDF file, 0.08 MB [file spectrum.03562-22-s0001.pdf]

SUPPLEMENTAL MATERIAL

SUPPLEMENTAL FIGURES

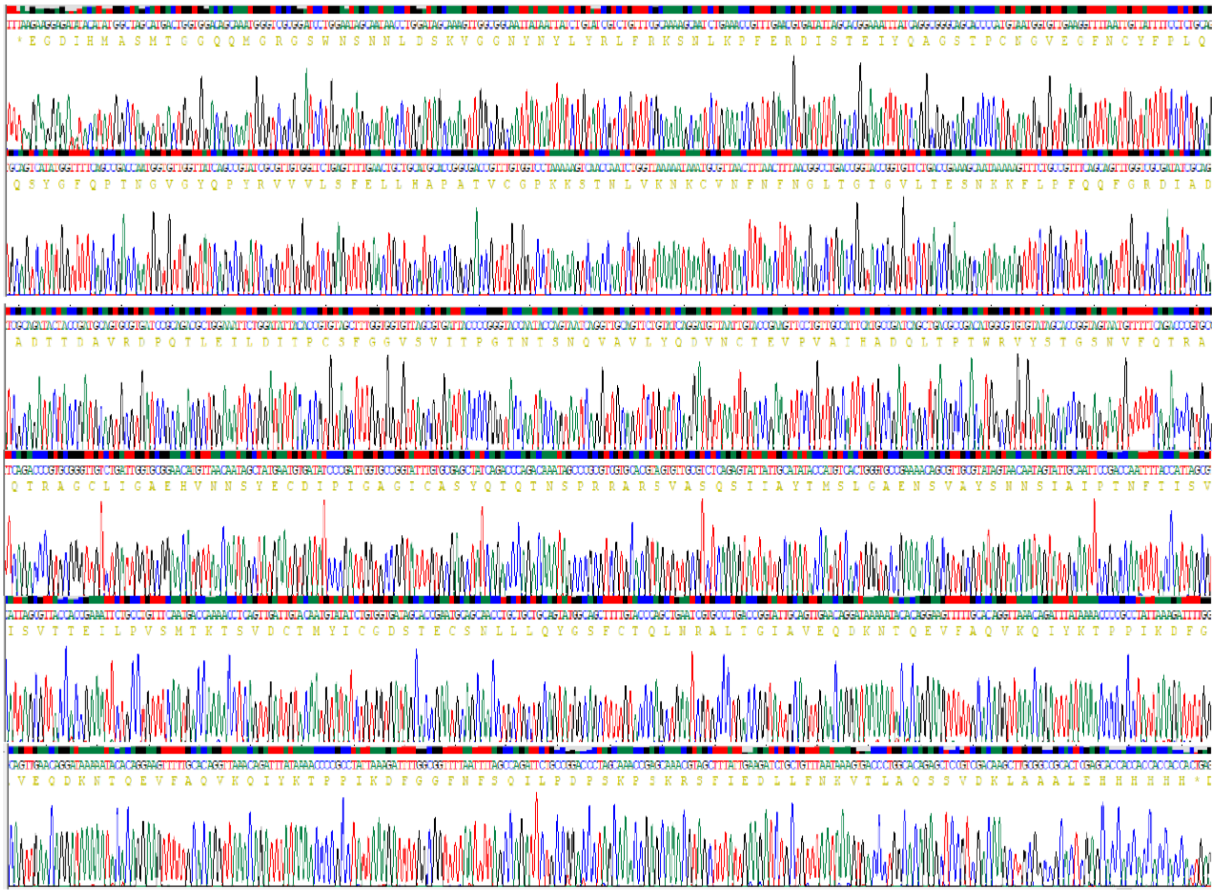

Fig. S1. Gene sequencing of reconstructed pET21a(+)/RBMFP plasmid.

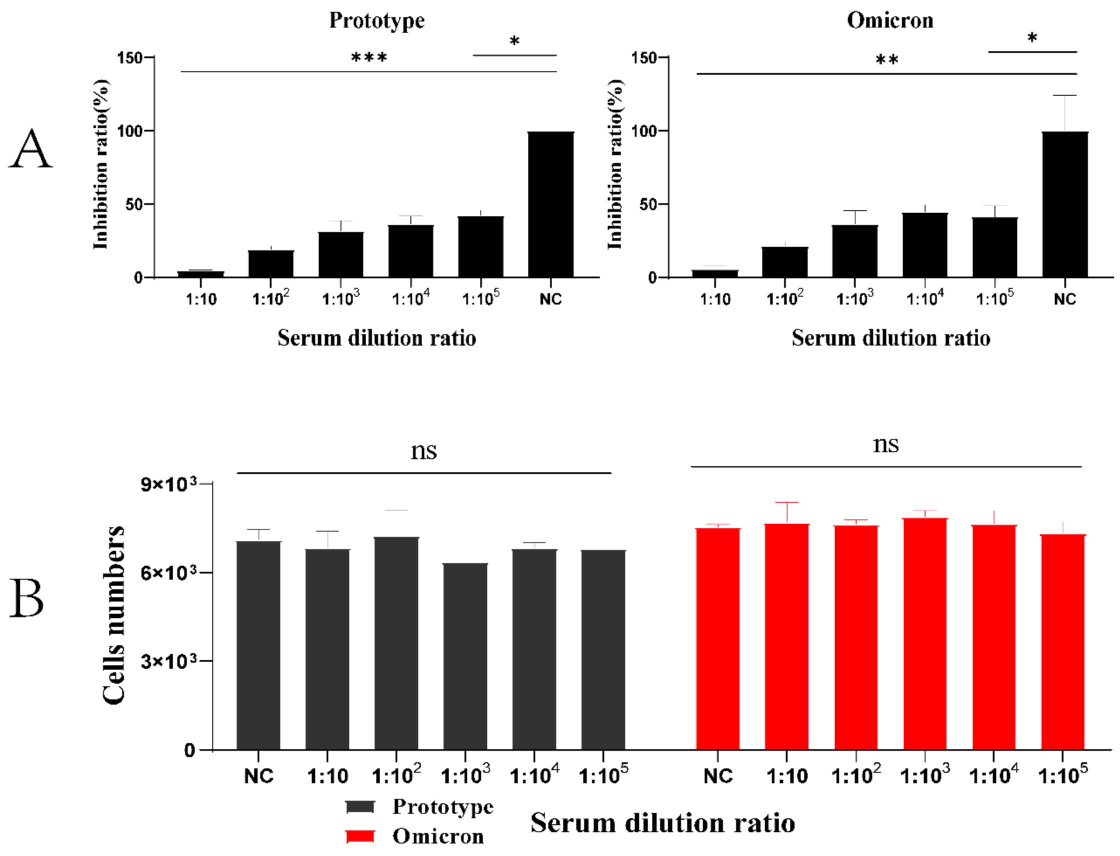

Fig. S2. Cell numbers counted and inhibition ratio of concentrations diluted of mice serum induced by RBMFP to neutralize SARS-CoV-2 pseudovirus (*P< 0.05, **P< 0.01, ***P < 0.001, ns: no statistical significance).

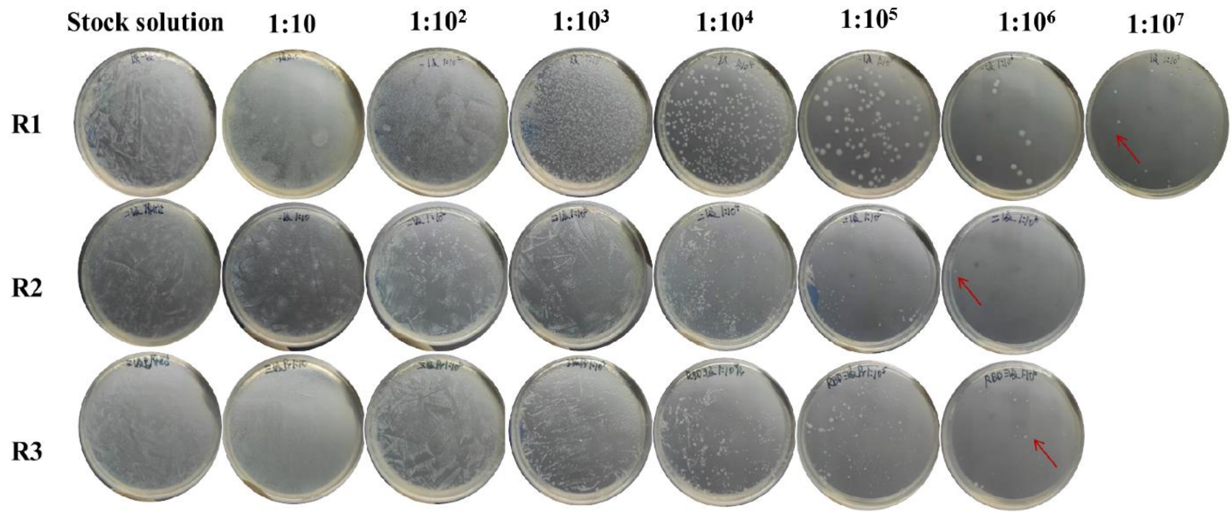

Fig. S3: The capacity of each round library after RBMFP protein panning. R1: The first round after panning. R2: The second round after panning. R3: The third round after panning. The red arrows show the monoclonal colonies on each plate, and the stock solution was the none diluted bacterial fluid from the last panned library. The numbers 1:10 to 1:10^7 were the dilution ratio of each library.

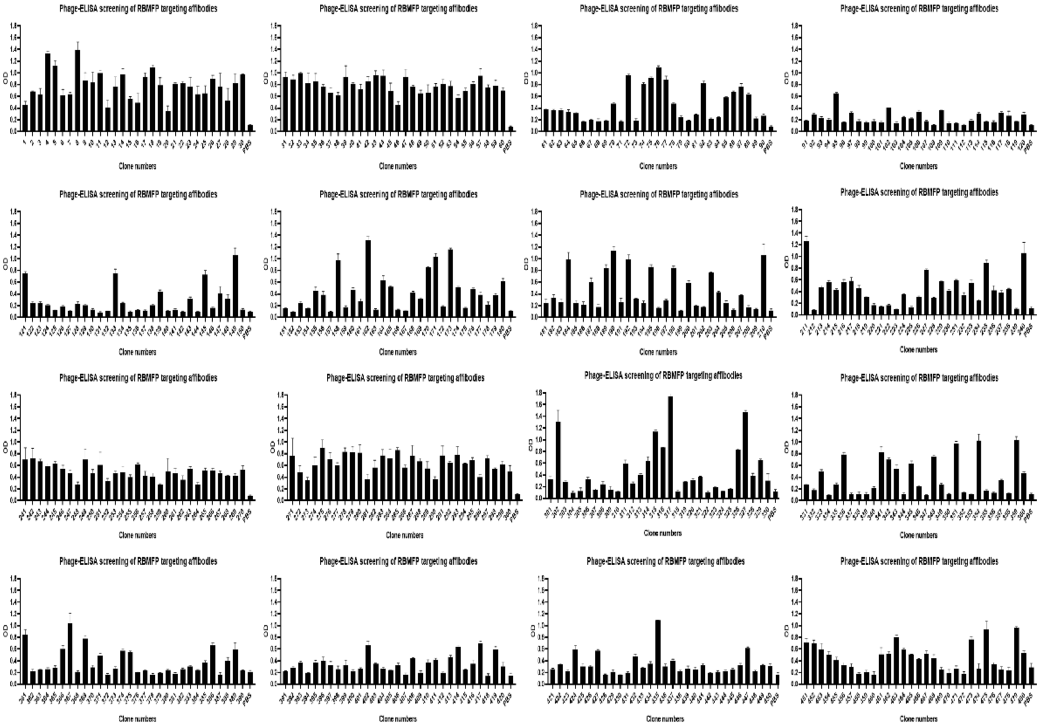

Fig. S4. 480 monoclonal strains chosen from the 3rd RBMFP targeting affibody library and selected by phage-ELISA.

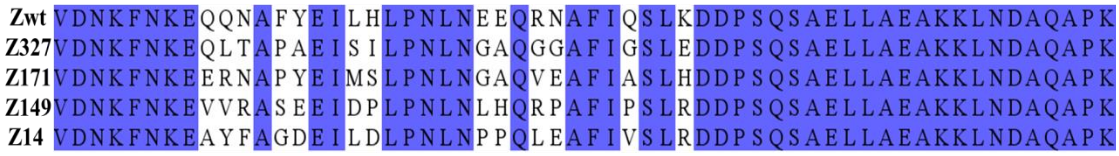

Fig. S5. Amino acid sequences of the top 4 affibody molecules selected targeting SARS-CoV-2 RBMFP protein. The mutation sites of affibodies are shown in white.

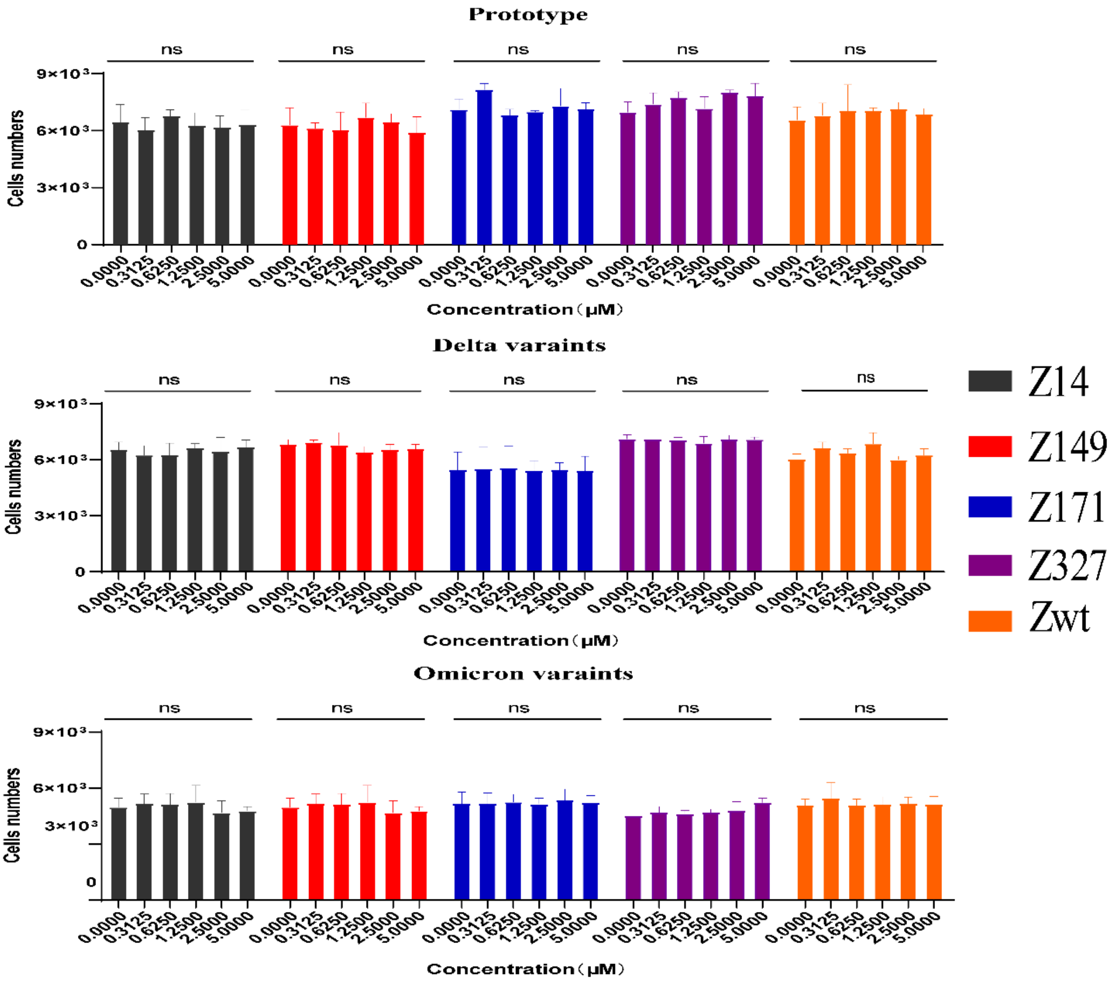

Fig. S6. Cell numbers counted in affibody neutralization to SARS-CoV-2 pseudoviruses (ns: no statistical significance).

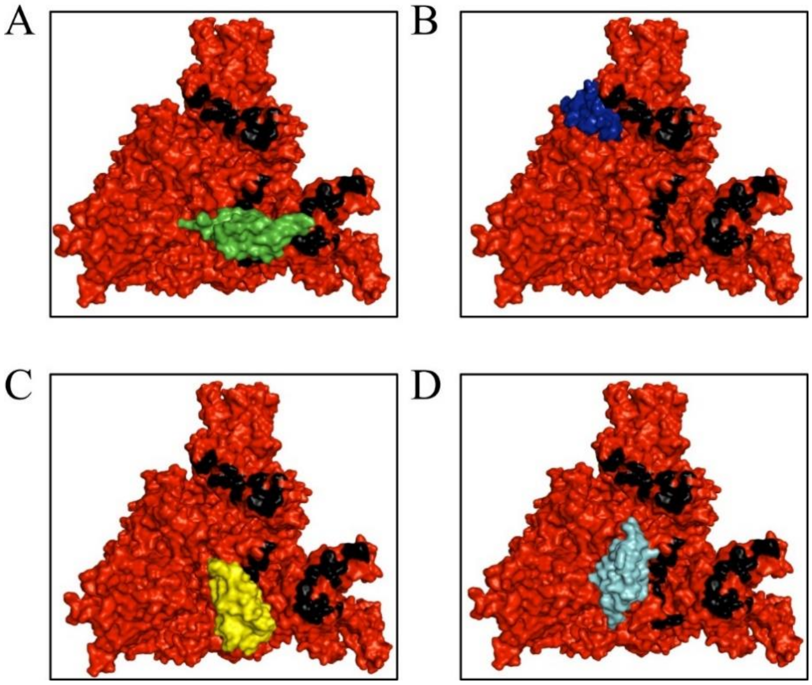

Fig. S7. Binding patches of Z14 (A), Z149 (B), Z171 (C), and Z327 (D) with SARS-CoV-2 spike protein (PBD: 7DWZ) shown in the surface model. Z14 (green) and Z171 (yellow) directly bind to the center of the area where spike protein (red) contact ACE2 (black) in Delta variants. Z149 (blue) and Z327 (cyan) bind near the center and limit their steric effect of halting virus infection in Delta and Omicron variants with D614G mutation.
